# Clonal Expansion of Macrolide-Resistant Sequence Type 3 *Mycoplasma pneumoniae*, South Korea

**DOI:** 10.3201/eid2408.180081

**Published:** 2018-08

**Authors:** Joon Kee Lee, Joon Ho Lee, Hyunju Lee, Young Min Ahn, Byung Wook Eun, Eun Young Cho, Hwa Jin Cho, Ki Wook Yun, Hoan Jong Lee, Eun Hwa Choi

**Affiliations:** Seoul National University College of Medicine, Seoul, South Korea (J.K. Lee, J.H. Lee, H. Lee, E.Y. Cho, K.W. Yun, H.J. Lee, E.H. Choi);; Seoul National University Children’s Hospital, Seoul (J.K. Lee, K.W. Yun, H.J. Lee, E.H. Choi);; Kangwon National University School of Medicine, Chuncheon, South Korea (J.H. Lee);; Seoul National University Bundang Hospital, Seongnam, South Korea (H. Lee);; Eulji University School of Medicine, Daejeon, South Korea (Y.M. Ahn, B.W. Eun);; Chungnam National University College of Medicine, Daejeon (E.Y. Cho);; Chonnam National University Medical School, Gwangju, South Korea (H.J. Cho);; Chonnam National University Children's Hospital, Gwangju (H.J. Cho)

**Keywords:** Mycoplasma pneumoniae, multilocus sequence typing, macrolides, drug resistance, bacteria, antimicrobial resistance, South Korea

## Abstract

To investigate the genetic background for the emergence of macrolide resistance, we characterized the genetic features of *Mycoplasma pneumoniae* using multilocus sequence typing. Of the 146 *M. pneumoniae* strains collected during the 5 consecutive outbreaks of *M. pneumoniae* pneumonia during 2000–2016 in South Korea, macrolide resistance increased from 0% in the first outbreak to 84.4% in the fifth. Among the 8 sequence types (STs) identified, ST3 (74.7%) was the most prevalent, followed by ST14 (15.1%). Macrolide-susceptible strains comprised 8 different STs, and all macrolide-resistant strains were ST3 (98.3%) except 1 with ST14. The proportion of macrolide-resistant strains in ST3 remained 2.2% (1/46) until the 2006–2007 outbreak and then markedly increased to 82.6% (19/23) during the 2010–2012 outbreak and 95.0% (38/40) during the 2014–2016 outbreak. The findings demonstrated that clonal expansion of ST3 *M. pneumoniae* was associated with the increase in macrolide resistance in South Korea.

*Mycoplasma pneumoniae* is one of the major causes of community-acquired pneumonia in children and adolescents (*1*). *M. pneumoniae* pneumonia develops with a gradual onset of constitutional symptoms over several days to a week (*2*). Although most patients may have self-limited symptoms resembling those of an upper respiratory infection, *M. pneumoniae* is recognized for producing a broad array of extrapulmonary manifestations that include hemolysis, rash, and joint involvement (*1*,*3*). Previous studies have established that the P1 adhesin (P1), a 170-kd surface protein located at the tiplike structure of virulent *M. pneumoniae*, mediates its cytadherence to the surface of respiratory epithelial cells, which is a critical step in the infection process (*4*).

Epidemiologic studies have shown that outbreaks of *M. pneumoniae* pneumonia occur every 3–7 years, varying from region to region on the basis of underlying low-grade endemic activity (*5*,*6*). Because the P1 is a major determinant of virulence, most studies have targeted the genetic variations of the *p1* gene to explain specific genotypes that link to the outbreaks (*7*–*9*). However, because *M. pneumoniae* has a small genome size, its genomic diversity is known to be limited among strains, and any associations of specific genotypes with disease outbreaks are rarely found (*7*,*10*,*11*). Since the first report of a macrolide-resistant *M. pneumoniae* isolated from a child in Japan in 2001 (*12*), several countries in Asia, including South Korea, Japan, and China, have reported increased prevalence of macrolide resistance (*13*–*16*). Point mutations in domain V of 23S rRNA are responsible for macrolide resistance. High antimicrobial consumption may provide selective pressure for the development of macrolide resistance (*13*). However, rapid dissemination of multiple clones that exhibit macrolide resistance also can lead to the increase in macrolide resistance rates in the community (*17*).

The multilocus sequence typing (MLST) scheme was first applied in *Neisseria meningitidis* and is a tool widely used for strain differentiation in many types of bacteria (*18*). Recently, Brown et al. developed an MLST scheme for *M. pneumoniae* using 8 housekeeping genes with a relatively high discriminatory ability (*19*). MLST has the potential to be used as a tool to characterize strains isolated during epidemic outbreaks of *M. pneumoniae* pneumonia and to investigate relatedness of specific genetic background to the emergence of macrolide resistance.

To clarify the genetic diversity of *M. pneumoniae* strains between outbreaks, we conducted an MLST analysis of *M. pneumoniae* detected from children in whom community-acquired pneumonia was diagnosed during 5 consecutive epidemics of *M. pneumoniae* pneumonia during 2000–2016 in South Korea. We also sought to find the genetic background that may explain the emergence of macrolide resistance among *M. pneumoniae* strains.

## Materials and Methods

### *M. pneumoniae* Strains

This study comprised *M. pneumoniae* strains detected from children with pneumonia at 5 hospitals during 5 consecutive outbreaks of *M. pneumoniae* pneumonia in South Korea: 2000, 2003–2004, 2006–2007, 2010–2012, and 2014–2016. Epidemic periods were previously defined by an interval spanning an increase of >5 cases/2 months over a 4-month period to a decrease of <5 cases/2 months over a 4-month period in the primary site of this study (*6*,*14*). *M. pneumoniae* pneumonia was diagnosed using the following criteria: 1) the presence of rales on auscultation or infiltration of the lung demonstrated on chest radiograph and 2) a positive PCR result for *M. pneumoniae* or isolation of *M. pneumoniae* on culture. Five hospitals participated in this study: Seoul National University Children’s Hospital (Seoul), Seoul National University Bundang Hospital (Seongnam), Eulji Hospital (Seoul), Chungnam University Hospital (Daejeon), and Chonnam University Hospital (Gwangju). Two hospitals in Seoul and 1 hospital in Seongnam cover the Seoul metropolitan area, where almost half of the South Korea population resides. Daejeon is representative of central South Korea, and Gwangju represents the south. *M. pneumoniae* strains detected from community-acquired cases were included for further analysis. We excluded healthcare-associated infections and intrafamilial infections.

### DNA Extraction from *M. pneumoniae* and Macrolide Resistance

We extracted DNA directly from the cultivated *M. pneumoniae* or from nasopharyngeal aspirates using an extraction kit (DNeasy Kit; QIAGEN, Hilden, Germany), according to the manufacturer’s instructions. We amplified the *p1* gene by PCR for the detection of *M. pneumoniae*. Starting in 2010, *M. pneumoniae* was cultivated using pleuropneumonia-like organism broth and agar for nasopharyngeal aspirates or pleural fluid obtained from the patient as previously described (*20*). The mutations responsible for macrolide resistance were confirmed by sequencing analysis of the amplified PCR products for domain V of the 23S rRNA gene. Primers MP23SV-F 5′-TAACTATAACGGTCCTAAGG-3′ and MP23SV-R 5′-ACACTTAGATGCTTTCAGCG-3′ were used, and PCR products were sequenced to identify mutations (*14*).

### MLST Analysis and P1 Typing

We performed MLST on the *M. pneumoniae* DNA samples as previously described. Each allele was assigned to the 8 housekeeping genes (*ppa*, *pgm*, *gyrB*, *gmk*, *glyA*, *atpA*, *arc*, and *adk*), and a corresponding sequence type (ST) was given for each sample (*19*). We submitted new alleles and allelic profiles to the PubMLST database for MLST assignment (http://pubmlst.org/mpneumoniae/). We used eBURST version 3 software (http://eburst.mlst.net/) to estimate the relationships among the strains and to assign strains to a clonal complex (CC) (*21*). We also conducted P1 typing for the samples from 2000–2016 by sequencing 2 of the repetitive elements located in the *p1* gene of *M. pneumoniae* genome: *RepMP2/3* and *RepMP4*. We assigned P1 subtypes and each subtype variant by comparison with previously published data (*22*).

### Statistical Analysis

We conducted statistical analysis using IBM SPSS Statistics for Windows version 23.0 (IBM Corp., Armonk, NY, USA). A linear-by-linear association model was used for Pearson’s χ^2^ test for trend analyses. We considered a p value of <0.05 as significant.

### Ethics Statement

The institutional review board of Seoul National University Hospital approved the study protocol (IRB no. H-1012–007–341). Informed consent was exempted because nasopharyngeal aspirates were obtained as a standard of patient care to identify the etiologic agents of acute lower respiratory tract infections.

## Results

### *M. pneumoniae* Strains and Macrolide Resistance

Our study comprised 146 *M. pneumoniae* DNA samples. Samples included for each outbreak were selected as follows: 21 samples from 2000, 14 samples from 2003–2004, 29 samples from 2006–2007, 37 samples from 2010–2012, and 45 samples from 2014–2016. Until the 2006–2007 outbreak, DNA samples were directly collected from respiratory samples (64 samples), and DNA samples from the 2010–2012 outbreak were extracted from cultured *M. pneumoniae* (82 samples).

For the samples before 2010, we included all the available specimens because of a limited number of archived samples relative to the 2010–2012 and 2014–2016 outbreaks. During the 2 outbreaks for which we have a larger number of samples, we selected samples to represent geographic region, month of isolation, and ages of patients. The proportions of selected samples were 28.5% (2010–2012) and 32.4% (2014–2016) of the archived samples. Of the study population, 56.1% were male. The mean age of children was 6.5 years; 7.4% were <2 years of age, 32.1% were 2–5 years of age, 60.5% were >5 years of age. The remaining 187 samples that were not selected for this study did not differ significantly from selected samples with respect to mean patient age and geographic region.

**Figure 1:**
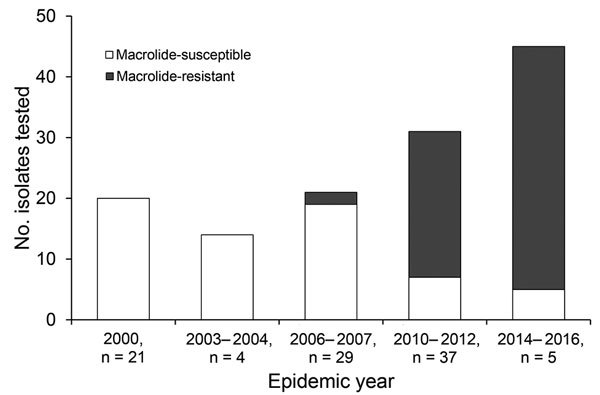
Macrolide resistance of *Mycoplasma pneumoniae*, South Korea, 2000–2016. Each number on the bar graph indicates the macrolide-resistancet of each epidemic year. The proportion of macrolide resistance strains by each outbreak were as follows: 0% (2000 and 2003–2004), 3.4% (2006–2007), 54.1% (2010–2012), and 84.4% (2014–2016).

From the 146 *M. pneumoniae* strains investigated, 59 (40.4%) strains expressed macrolide resistance associated with either an A2063G (58 [98.3%]) or A2064G (1 [2.7%]) mutation in the 23S rRNA gene. Differences in macrolide resistance were recognized in each of the outbreaks (Figure 1). Strains from the 2000 and the 2003–2004 outbreaks were all susceptible to macrolide. The proportion of macrolide-resistant strains for each outbreak was 3.4% for 2006–2007, 54.1% for 2010–2012, and 84.4% for 2014–2016. The trend analysis for macrolide resistance acrss the 5 periods showed a significant increase (0% to 84.4%; p<0.0001).

### MLST and P1 Typing of *M. pneumoniae*

**Figure 2:**
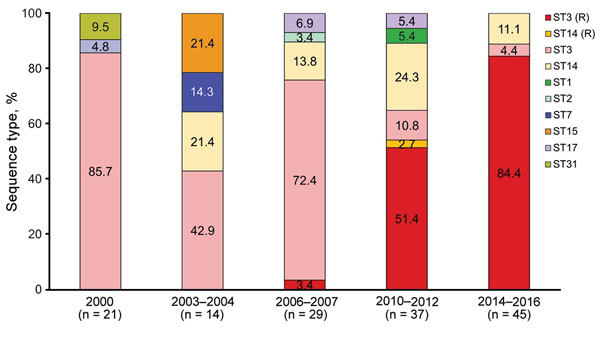
*Mycoplasma pneumoniae* ST distribution by each outbreak and macrolide resistance within specific STs, South Korea, 2000–2016. Each number of the box indicates proportion of each ST. (R) designates macrolide resistance. ST, sequence type.

MLST analysis identified 8 STs during the study period: ST1, ST2, ST3, ST7, ST14, ST15, ST17, and ST31 (Table 1). The epidemic distribution of STs is shown in Figure 2. During the study period, ST3 (109 [74.7%]) was the most commonly identified ST, followed by ST14 (22 [15.1%]). ST3 was also the predominant ST found during all 5 outbreaks. A total of 3–5 STs circulated during each outbreak, and several minor STs (ST7, ST15, and ST31) that circulated in the earlier outbreaks were not found in the recent outbreaks. The distribution of ST did not differ by geographic region.

**Table 1:** *Mycoplasma pneumoniae* STs and allelic profile of each ST with corresponding P1 type, South Korea, 2000–2016*

ST	No. (%) isolates	Allelic profile								P1 type
*ppa*	*pgm*	*gyrB*	*gmk*	*glyA*	*atpA*	*arcC*	*adk*
ST1	2 (1.4)	1	2	1	1	1	3	2	1	1
ST2	1 (0.7)	2	3	2	2	2	4	1	1	2b
ST3	109 (74.7)	1	2	1	1	1	3	1	1	1
ST7	2 (1.4)	2	3	2	2	2	4	1	2	2
ST14	22 (15.1)	2	3	2	2	4	4	1	5	2a, 2c
ST15	4 (2.7)	2	3	2	2	4	4	1	1	2a
ST17	4 (2.7)	1	5	1	1	1	3	1	1	1
ST31†	2 (1.4)	2	8	2	2	2	4	1	1	2

*ST, sequence type. †Newly identified ST in this study.

We conducted P1 typing for 85 strains from 2000–2016. Overall, we identified 5 subtypes and subtype variants of P1 (1, 2, 2a, 2b, and 2c). P1 subtype 1 was the main subtype at 70.6% (60 strains), followed by subtype 2 with 29.4% (25 strains). P1 subtype 1 accounted for 85.7% of the 2000 outbreak, 42.9% in 2003–2004, 75.0% in 2006–2007, 71.9% in 2010–2012, and 50.0% in 2014–2016. P1 subtypes 2 and 2a were observed up until the 2003–2004 outbreak, and subtype variant 2c was observed from the 2006–2007 outbreak. P1 subtype variant 2c (13 [52.0%]) was most common within P1 subtype 2, followed by subtype variants 2a (7 [28.0%]), 2 (4 [16.0%]), and 2b (1 [4.0%]). Each ST was associated with a single P1 subtype or subtype variant, except for ST14 strains, which possessed both P1 subtype variants 2a and 2c.

### Sequence Type and Macrolide Resistance

Macrolide-susceptible strains consisted of 8 different STs identified. Among 8 STs, ST3 (58/109 [53.2%]) and ST14 (1/22 [4.5%]) were the only ones that expressed macrolide resistance (Table 2). One ST14 strain that expressed macrolide resistance was from the 2010–2012 outbreak and harbored the A2063G mutation.

**Table 2:** Distribution of *Mycoplasma pneumoniae* STs by macrolide susceptibility, South Korea, 2000–2016*

ST (no. isolates)	No. (%) isolates	
Macrolide-susceptible	Macrolide-resistant
ST1 (2)	2 (2.3)	0
ST2 (1)	1 (1.1)	0
ST3 (109)	51 (58.6)	58 (98.3)
ST7 (2)	2 (2.3)	0
ST14 (22)	21 (24.1)	1 (1.7)
ST15 (4)	4 (4.6)	0
ST17 (4)	4 (4.6)	0
ST31 (2)	2 (2.3)	0
Total (146)	87 (100)	59 (100)

*ST, sequence type.

We found a correlation between the increasing proportion of macrolide resistance and the proportion of macrolide-resistant strains within ST3. All of the strains in ST3 were macrolide susceptible up ntil the 2003–2004 outbreak, and only 1 of the 22 strains in ST3 showed macrolide resistance in the 2006–2007 outbreak. The proportion of macrolide-resistant strains within ST3 dramatically increased to 82.6% (19/23) during the 2010–2012 outbreak and to 95.0% (38/40) during the 2014–2016 outbreak (Table 3). These data strongly suggest that a clonal expansion of ST3 *M. pneumoniae* is responsible for the increasing proportion of macrolide resistance.

**Table 3:** Distribution of macrolide susceptibility within *Mycoplasma pneumoniae* sequence type 3, South Korea, 2000–2016

Macrolide susceptibility	Epidemic years, no. (%) strains				
2000	2003–2004	2006–2007	2010–2012	2014–2016
Susceptible	18 (100)	6 (100)	21 (95.5)	4 (17.4)	2 (5.0)
Resistant			1 (4.5)	19 (82.6)	38 (95.0)
Total	18	6	22	23	40

### eBURST Analysis

**Figure 3:**
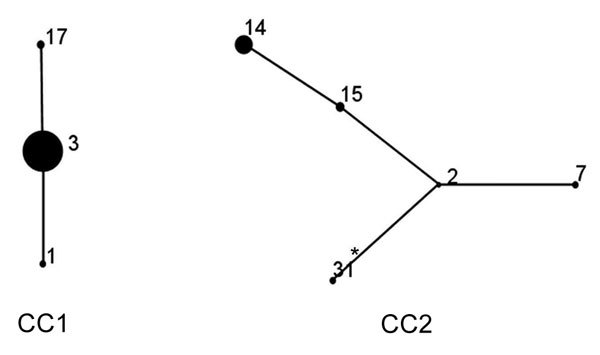
*Mycoplasma pneumoniae* sequence type (ST) relationship of 146 strains by eBURST analysis (http://eburst.mlst.net/), South Korea, 2000–2016. Two main CCs were defined without any singleton. ST3 and ST2 were the predicted founder of each CC. Numbers on the diagram correspond to STs. The size of each circle correlates with the number of isolates of each ST. CC, clonal complex.

Two CCs were identified on eBURST analysis (Figure 3). CC1 contained 115 (78.8%) strains with 3 STs, and CC2 contained 31 (21.2%) strains with 5 STs. ST3 and ST2 were predicted to be the founder ST for each CC. The newly identified ST in this study was ST31, which was part of CC2 and a single-locus variant of ST2.

## Discussion

In this study, molecular microbiological analysis of MLST found that the increase of macrolide resistance in South Korea during a 17-year period (2000–2016) was related to changes in genetic backgrounds of *M. pneumoniae* strains. Traditionally, diagnosis of *M. pneumoniae* pneumonia relied on the increase of mycoplasma antibody or the presence of IgM. However, with the emergence of macrolide resistance, it became important to know the presence of macrolide resistance of *M. pneumoniae*. Thus, direct detection of *M. pneumoniae* either by culture or by PCR is crucial for testing macrolide resistance. Although no treatment strategy for macrolide-resistant *M. pneumoniae* pneumonia has yet been established, alternative antimicrobial drugs (tetracyclines and fluoroquinolones) can be considered when patients remain febrile at least 48–72 hours after macrolide treatment (*23*,*24*).

The whole genome of *M. pneumoniae* is ≈820 kb and has up to 700 coding operons. On the basis of the data of the comparative analysis of 58 strains, the genome appears to be highly conservative among the strains (*1*). Because of relatively low sequence variations and many repetitive elements within the genome of *M. pneumoniae*, epidemiologic investigations on genetic diversity have focused on the sequence variations of *p1* gene. Several studies explored an association between P1 subtypes and epidemic outbreaks. The postulation was based on the idea that development of temporary immunity to 1 type by an outbreak might enable reemergence of the other (*7*). Despite experimental grounds and the scientific reasoning, studies that followed did not support the hypothesis. A study from Germany that examined the P1 molecular typing of 467 *M. pneumoniae* did not support predominance of 1 of the 2 major P1 subtypes or switching of the subtypes during the endemic situation before and during the outbreak period (*10*). Furthermore, Diaz et al. examined 199 *M. pneumoniae* samples from 17 investigations of cases, small clusters, and outbreaks that were supported by the Centers for Disease Control and Prevention (Atlanta, GA, USA) to determine the association of P1 subtypes with macrolide resistance (*11*). In that study, the distribution of P1 subtypes did not differ between macrolide-resistant and macrolide-susceptible *M. pneumoniae* strains, suggesting that an individual P1 subtype is not associated with macrolide-resistant genotype.

Molecular typing methods other than P1 typing have attempted to discriminate strains in each outbreak and to find correlations between strain diversity and macrolide resistance of *M. pneumoniae*. Recent studies use sophisticated technologies such as quantitative PCR for the diagnosis and multilocus variable-number tandem-repeat analysis (MLVA) for characterization and classification. Waller et al. reported 7 different MLVA profiles associated with certain P1 subtypes from 12 *M. pneumoniae* strains during an outbreak in the United States (*25*). MLVA and MLST also were adopted for studying *M. pneumoniae* in recent years (*19*,*26*). MLVA uses naturally occurring variations in the number of tandem repeated DNA sequences found in many different loci of the genome. MLST characterizes the isolates of microbial species using DNA sequences from internal fragments of multiple housekeeping genes. Of the 2 molecular typing methods, the discriminatory power of MLST scheme with the 8 loci was 0.784 for the collection of 57 isolates, whereas MLVA scheme was 0.633 (*19*,*27*). This finding was due partly to removal of the *Mpn1* locus in MLVA scheme because of inconsistency in nomenclature and identification of repeat regions (*28*).

Sun et al. reported that the rates of resistance mutations increased in parallel with an increase in MLVA type 4572 during 2003–2007 and 2008–2013 and decreased in parallel with a decrease in type 4572 during 2014 and 2015, based on 480 *M. pneumoniae* isolates from children in Beijing, China, during 2003–2015 (*29*). A study of MLVA typing of *M. pneumoniae* strains isolated during 2004–2014 in Yamagata, Japan, reported that the prevalence of macrolide resistance–associated mutations in type 4572 was 59.7% (108/181), which was significantly higher than in other MLVA strains (*30*). The prevalence of the A2063G mutation in type 4572 strains was 0.9% (1/107) during 2004–2010 but became 83.8% (62/74) during 2011–2014. A recent study from Hong Kong reported an increased prevalence in macrolide resistance as well and identified type 4572 strain as the contributor (*31*). In that study, the authors reported that the macrolide resistance rate for type 4572 significantly increased from 25.0% in 2011 to 100% in 2014. In contrast to those studies, a study of 152 *M. pneumoniae* strains conducted by Liu et al*.* suggested that macrolide-resistant strains were multiclonal origin (*17*). The results of that study clustered 137 macrolide-resistant strains into 15 MLVA types, indicating the high rate of macrolide resistance could result from dissemination of the multiple resistant clones. This conflicting result might have resulted from the 5 loci MLVA, which applied the earlier MLVA method, including the unstable *Mpn1* locus (*26*).

We cannot, at this point, answer with confidence why an ST3 strain became the most prevalent strain among macrolide-resistant *M. pneumoniae*. We can, however, speculate. Mutation or some other mechanism could have caused the previously macrolide-susceptible ST3 strains to become macrolide resistant, and the new strain could have developed an ability to disseminate through high-density populations. Antimicrobial selective pressure could have aided this development. An alternative possibility is that the macrolide-resistant strains were introduced to and spread rapidly through the community. Our data demonstrate that ST3 and ST14 are not genetically related; they share 1 of 8 allelic loci and differ in P1 subtypes. Analysis with eBURST shows they exist in different clonal complexes. Further research with whole-genome sequencing can reveal the distinguishing characteristics of macrolide-resistant and -susceptible strains within ST3 strains (*32*,*33*). In addition, whole-genome sequencing may reveal how the macrolide-resistant ST3 became predominant.

Antimicrobial pressure would have played a role to some extent because South Korea is a high antimicrobial drug use country. Trend analyses of the national data on antimicrobial drug consumption (expressed in defined daily doses [DDD]/1,000 inhabitants/day [DID]) demonstrated an increase in macrolide use in the community during 2005–2014 (*34*). Macrolide use remained steady until 2007; however, DID increased consistently from 2007 (2.59 DID) through 2014 (4.14 DID). In particular, in children <6 years of age, the increase measured from 7.73 DID (2007) to 9.51 DID (2014), with a peak of 11.99 DID in 2011. Therefore, the increase in macrolide consumption might explain in part the 17-year (2000–2016) change of macrolide resistance in *M. pneumoniae*. Nevertheless, the expansion of a single clone, which was demonstrated by our study, makes us assume another possible explanation. We think the macrolide-resistant clone, which we characterize as an ST3 clone by MLST, might have been introduced before the 2010–2012 outbreak and spread extensively among the community where the population density is high, supported by antimicrobial selective pressure.

Although various studies have investigated the genetic association of *M. pneumoniae* with epidemic outbreaks or macrolide resistance, research using MLST is currently insufficient. Our study demonstrates the predominance of ST3 throughout the entire study period. ST14 was the second most common ST found in all of the epidemics, except for the 2000 epidemic year, implying that 2 clonal complexes and their STs have existed simultaneously. As for ST and macrolide resistance, our study demonstrates ST3 as a major ST that harbors macrolide resistance and found a single macrolide-resistant ST14 strain. Until the 2006–2007 outbreak, most of the ST3 strains were macrolide susceptible, but ST3 from outbreaks since 2010–2012 showed macrolide resistance >90% on an increasing tendency. This finding confirms the concept that the increased prevalence of macrolide resistance is related to a single clone expansion. The results of our study can be compared with previous studies using MLST and MLVA. Brown et al. demonstrated an association among MLVA type 4572, P1 subtype 1, and CC1 (ST1, ST3, ST5, ST9, ST11, and ST12) (*19*). Our study also shows a relationship between P1 subtype 1 and CC1 (ST1 and ST3), which is consistent with their report. This relationship could have occurred by acquisition of 23S rRNA mutation within certain STs from predisposing genetic factors or by an introduction of a macrolide-resistant strain. On the basis of the studies we have described and our own results, clonal expansion of certain molecular types, 4572 in MLVA and ST3 in MLST, is likely to be the reason for the marked increase in macrolide resistance.

Our study has several limitations. First, even though *M. pneumoniae* strains were collected from 5 hospitals, the data might not represent reality nationwide. However, this was the best possible multicenter-based study we could devise, given that no nationwide surveillance system exists. Second, despite our best efforts, the numbers of strains in the earlier outbreaks were smaller than those collected in the later outbreaks, when culture was performed prospectively. However, this study is of value because the distribution of STs in a certain region for a relatively long period of time is well described. The finding that an expansion of a single ST contributed to the increase in macrolide resistance is a potentially powerful insight for further research. Additional studies that investigate the epidemiology and mechanism of acquiring macrolide resistance will give further insight into better treatment strategies. In particular, further studies should be addressed to in silico methods for the analysis of genetic background that can explain macrolide resistance within ST3 strains. Recent advances in microbiology have made whole-genome sequencing a valuable investigation tool that can lead to the identification of a specific genotype associated with macrolide resistance or virulence of *M. pneumoniae*.

In summary, we found that, during outbreaks of *M. pneumoniae* pneumonia that showed substantial increase in macrolide resistance in South Korea, all but 1 macrolide-resistant strain was ST3. These findings demonstrate that clonal expansion of an ST3 *M. pneumoniae* was associated with the increase in macrolide resistance in South Korea.
